# Opportunities in Health Education in the Post-COVID-19 Era: Transforming Viral to Vital

**DOI:** 10.7759/cureus.30371

**Published:** 2022-10-17

**Authors:** Andreas Gerostathis, Eleftheria C Economidou, Dimitra Mpousiou, Paraskevi Katsaounou, Elpidoforos S Soteriades

**Affiliations:** 1 Internal Medicine, Istiaia Primary Healthcare Center, Istiaia, GRC; 2 Medicine, University of Ioannina, Ioannina, GRC; 3 Pulmonology, Daphne Health Center, Athens, GRC; 4 Pulmonary and Critical Care Department, Evaggelismos Hospital, National and Kapodistrian University of Athens, Athens, GRC; 5 School of Economics and Management, Healthcare Management Program, Open University of Cyprus, Nicosia, CYP

**Keywords:** school programs, health education, vital, viral, education, covid-19 pandemic

## Abstract

Although the current pandemic is associated with many difficulties and social challenges, in parallel, it has been linked with new opportunities. The field of education and, in particular, health education, represent a highlighted example. According to the United Nations Educational, Scientific and Cultural Organization (UNESCO) statistics, the education of more than 1.5 billion students in 188 countries around the globe was affected due to the closure of educational institutions following the coronavirus outbreak. In the present study, we examine the development of possibilities, prospects, and opportunities in the post-COVID-19 era in the field of health education. Using reflective observations on what we have gained as knowledge during this pandemic, we summarize five VITAL aspects of health education: the emerging value in health; the power of preventive interventions in health education; the transmission of health messages by students in the context of communication between school, family, and community; the exploitation of contemporary e-learning applications as a mixed hybrid learning mode; and the life examples as projected from the theoretical principles of health education to real scenarios. In conclusion, the global pandemic crisis, serving as a "violent teacher," presents us with hidden potential, promising new prospects in the field of health education that we need to exploit.

## Editorial

Introduction

Coronavirus disease 2019 (COVID-19) represents a new infectious disease caused by the severe acute respiratory syndrome coronavirus 2 (SARS-CoV-2) that has led to a pandemic and caused a universal concern [1]. The extraordinary condition of the pandemic and the need to adapt to the SARS-CoV-2 challenges resulted in significant costs to physical and mental health around the world [1-2], which increased associated morbidity and mortality in all ages. Furthermore, the unprecedented climate of fear, isolation, minimization of social interactions, and disruption of people’s daily routines changed the way in which all manner of activities were conducted, including education, work, social interaction, and hobbies for most people [3]. As a consequence, a number of health problems arose, including physical, psychological, social, and emotional.

In particular, education was a major field affected by the pandemic. The true extent of the impact on teaching and learning, for students and teachers alike, is yet to be fully determined [4]. During the pandemic, more than 80% of students around the world have been affected by school closures [5]. According to the United Nations Educational, Scientific and Cultural Organization (UNESCO), partial- or full-school closures were still affecting about 70% of the worldwide student population one year into the pandemic, and literacy levels were projected to decrease, with more than 100 million additional children affected. The more pessimistic scenarios refer to a looming "generational catastrophe" [6].

Balancing the above, we believe that there are several take-home messages that need to be recognized in an attempt to revive society’s systemic approach. Educators have a responsibility to promote fundamental values and principles, while education is closely linked to health and well-being and is considered one of the most important modifiable social determinants of health [5,7]. Many countries have tried to keep schools open or re-open schools safely in the event of prolonged closures by putting in place locally adapted health and safety policies to protect learners and school staff [7]. The changes in the realm of education, and more specifically, preventive interventions in the context of health education, have drawn attention to difficulties and obstacles, but have also given prominence to new possibilities and opportunities.

In the current manuscript, we analyze five important aspects of the above-described landscape by exploring the lessons learned from the current pandemic while focusing on the opportunities lying ahead in school activities and interventions in order to further develop the important field of health education. We discuss five specific possibilities that have been highlighted and could serve as a symbolic transformation from VIRAL to VITAL in health education, which include: value in health, interventions in health education, transfer and diffusion of health messages, applications of online and distance learning, and life examples: from vague theory to real life. Each opportunity has been aligned with one relevant excerpt from ancient Greek literature.

Aspect 1: Value in health

"Health is best": The great Greek philosopher Plato argues that health is one of the best goods [8].

The threat, danger, and uncertainty of the pandemic have shifted society’s focus on the values of life, health, and safety, which are recognized as valuable goods and demand particular attention, as opposed to the prevailing common perception that they are self-evident. In the famous pyramid of the hierarchy of human needs designed by the psychologist Abraham Maslow (1943: A Theory of Human Motivation), the scientist placed security at the base, as a fundamental good along with the main needs of survival [9]. However, the attribution of value to health is not self-evident. A large percentage of people, up to 40%, do not rank health in the top five values, giving priority to others, including the utmost value of freedom [10]. However, the value attributed to health is an important predictor of a person's intentions and behaviors [11-12], with the consequence that people adopt preventive behaviors only when they value health as a major asset. In other words, the more one considers health as a fundamental and primary need, the more one classifies it in its top values, the less one passes it as a given and self-evident, and the more one follows behaviors that protect and promote it [13].

The post-pandemic shift in social interest toward safety and health has consistently placed much higher importance on the strengthening, implementation, and adoption of preventive behaviors. As a consequence, several opportunities have emerged during the current pandemic regarding the value of health, which may be exploited in health education. Briefly, we may point out the reorganization of the human value system leading to the recognition of health as a primary asset and its placement in the center of people's interests; the reconstruction of the common prevailing perception that health is given and self-evident; the focus on safeguarding, defending, and strengthening health; and the need to promote, enforce, and implement preventive behaviors that protect health.

Aspect 2: Interventions in health education

"Best to prevent than treat" The Father of Medicine, Hippocrates, claims that prevention is better than treatment [14].

Interventions in the framework of health education constitute a widely promoted goal in public health. Activities and interventions are a vital component of campaigns to raise awareness and inform not only the students but also the general public. It is of primary importance to raise awareness and inform the student population within the supportive environment of schools, an ideal setting to communicate with a large number of young people [15]. Such actions aim to promote health-positive attitudes among students by improving knowledge, rebuilding perceptions, changing beliefs, modifying misconceptions, prioritizing values, and reinforcing attitudes that value health.

The global health crisis highlighted and reaffirmed the value and usefulness of health education, with particular attention to personal hygiene, fresh air, healthy breathing, a balanced diet, physical activity, and quality of sleep. The children were asked to use their knowledge and skills about proper hand washing, covering their mouths in coughing and sneezing, using clean tissues, and many more hygiene measures in real-life scenarios [16]. This acquired knowledge was applied along with new ones concerning the more frequent washing of hands, the avoidance of finger contact with the face, the eyes, or the mouth, and the observance of social distance. Never before have the goals of preventive interventions in schools been served more effectively and proved to be so relevant and necessary to everyday activities [17]. In addition, the need for additional actions related to the expression of negative emotions, management, and control of anxiety, dysthymia, tension, stress, anxiety, fear, panic, or anger, which strongly manifested during the period of pandemic under the state of global threat, has emerged [18]. As a result of all of the above, emerging opportunities include the necessity of continuing and enhancing preventive interventions; the importance of health promotion as an educational priority; and the enrichment of school interventions by including stress and negative emotion management modules through the cultivation and strengthening of personal and social skills [19-20].

Aspect 3: Transfer and diffusion of health messages

"We believe, the children, are the soul of the state." The wise ancient Greek legislator Solon claimed that children are the soul of each society.

As discussed below, children may also serve as vehicles for transferring ideas and information between the family and the greater community. This is important since public health and health education are closely interconnected as public health is considered the broadest bridge between science and society [18,21]. The current health "emergency" has made it clear that the effective transmission of health messages concerning universal measures to protect public health is a necessary precondition. According to the science of communication, extremely important elements are the content of the message, its expression, and the strengthening of its persuasiveness (exercise of persuasion) [22]. While it seems that parents are the ones who usually influence their children, the opposite may also be true, that is, children can be the ones who can influence the knowledge, attitudes, and behaviors of parents, but also their immediate community [23].

In important health-education issues, such as adhering to the no-smoking rule at home, children seem to play an effective role as counselors and facilitators in the transmission of anti-smoking messages to the home environment [24] as well as serving as educators of their peers in peer-to-peer activities [25-26]. Challenges, as well as opportunities, arose regarding the dissemination of health education messages through the active role of students who could act as transport vehicles outside the narrow confines of the classroom. Therefore, students could mobilize their parents, siblings, and other important adults by serving as catalysts, proving that their voice has power and deserves to be heard [24].

In such a way, the dynamics of the school could be expanded to achieve, through students, a new potential to serve as a no-barrier two-way multi-modal institution to promote communication and influence families and the wider community. The pandemic has forced us to make the connection between health education and the learner’s home as well as the wider society. Highlighted opportunities include the possibility of communication between the school and the students’ families and the wider community for the diffusion of health messages beyond the school hall and the implementation of a simple and promising practice of transmitting health-education principles via students to home environments and communities alike, serving as a new legacy for the future.

Aspect 4: Application of online and distance learning

"The need is an invincible strength." The great dramatic poet Aeschylus emphasizes that the power of need is invincible [27].

Over the last decade, digital tools in general and virtual learning environments, in particular, became increasingly prominent in education, but for the most part, they did not replace in-person learning [28]. During the pandemic, the number of online classes skyrocketed on a global scale, with more than 1.5 billion students in 188 countries around the globe being involved [29]. However, little consideration was given to whether online distance learning (ODL) is equivalent and has a similar cost-benefit profile to in-person education [4,29]. As discussed below, the benefits of ODL do not seem to outweigh the costs. It should be noted, though, that ODL is not equivalent to the use of digital technologies in education. Digital technology can be used even during in-person learning, and it is necessary for students to familiarize themselves with digital methods of searching for information and obtaining self-acquired knowledge. The implementation of e-learning educational activities, incorporated under the pressing necessity of the pandemic, offered, in addition to knowledge, a reminder of life and continuity, which we can use, especially when addressing students of the online generation but also at older ages.

Among the advantages of ODL, we may quote that most of the terms (online learning, open learning, web-based learning, computer-mediated learning, blended learning, m-learning, etc.) provide the ability to use a computer connected to a network, which offers the possibility to learn from anywhere, anytime, at any pace, and with any means [30]. Online learning is defined as "learning experiences in synchronous or asynchronous environments using different devices (e.g., mobile phones, laptops, etc.) with internet access" [31]. There is a plethora of programs and applications which function as platforms for ODL, and they may be used in formal as well as informal education. Such technology enhances accessibility, can reach into rural and remote areas, and increases the potential for individualized instruction, while also providing flexibility and comfort [31-35]. As far as flexibility is concerned, learners can learn anytime and anywhere, as they can plan their time for the completion of courses and recorded lectures available online [32-33]. Furthermore, these applications are cheap and easy to use, while students and teachers can also save time and money by avoiding transportation, accommodation, and the overall cost of institution-based learning [31-35]. Finally, ODL contributes to the preservation of cohesion within the class in times when state restrictions do not permit in-person learning. Students have the right to continue their education in the light of potential new states of emergency, as was the case of the coronavirus outbreak [29].

However, several disadvantages of ODL may also apply and need to be considered. ODL requires familiarity with the specific technology being used. It focuses on the cognitive component and the transfer of information while diminishing the role of emotional stimuli and the possibility to convert information into experience. Furthermore, ODL is associated with emotions of isolation and loneliness, and it makes learners’ attention more susceptible to distractions. There are several indications that it is less effective, but this requires additional long-term evaluation [4,18,32-33,36-37]. Effective teaching does not depend only, or even mainly, on the quality of the information. The way in which information is transferred is as important as the information itself. This depends on the way in which the teacher expresses himself and addresses the students [38-39]. More specifically, the COVID-19 pandemic has impacted the education of healthcare professionals, which relies on various sources of learning from teachers, peers, and patients [6]. Variations in eye contact, voice intensity, voice pitch, the pace of speech, body language, the atmosphere within the classroom, and the sense of group cohesion are all significantly damped down with ODL [40-42]. Furthermore, interactive learning opportunities, such as the ability of students to assume active roles, participate in live dialogue and discussions, the expression of doubt and disagreement, and the opportunity to debate, argue, and express thoughts and opinions, are all achieved to a lesser degree with ODL. Students are encouraged to participate more when they are being taught in person [32,43]. Passive listening is not as effective in achieving the desired learning outcomes since learning is influenced by an interaction of personal, behavioral, and environmental factors [44]. As such, the educational process has the teacher-learner relationship at its center [45].

ODL mainly focuses on information as its nature severely limits the ability to learn behaviors and instill values, which, according to Bandura’s Social Learning theory, are learned through the process of observation and emulation of role models in the environment [46-47]. Thus, online distance learning with social isolation is even more disadvantageous at younger ages. Finally, it limits the ability of the teacher to gain real-time feedback from students, which is a crucial factor in improving effectiveness and promoting motivation [32,44]. We always keep in mind that the center of every educational process is the human relationship between teacher and student [18].

Take-Home Message

The experience of the pandemic can function as a motivator to introduce further preventive interventions in health education, especially via online platforms, since this form of education was imposed in many parts of the world as part of the response to the pandemic. However, the scientific literature and our common experience show that the benefits of traditional in-person learning outweigh the advantages of ODL, which can nevertheless have a complementary role in cases where in-person learning is not physically possible or too costly [48]. Opportunities in this aspect include the possibility of using e-learning as an educational tool and a springboard for the utilization of internet applications in preventive interventions in the context of health education; the recognition of the need for improvements in online distance learning in terms of enhancing the interaction, feedback, and participation of learners while reducing cognitive load; and the exploration of the hybrid learning mode, by combining classical personal learning with e-learning activities, which seems to be more attractive and acceptable to students.

Aspect 5: Life examples: from theory to real scenarios

"Deeds and not words." The ancient Greek poet Aeschylus says that deeds are those that have value, not words [27].

What we teach in schools in the context of health education refers to protecting students from future hazards that seem distant, uncertain, vague, and frequently underestimated by children, due to their lack of perception of vulnerability and sense of omnipotence. Young people generally feel strong, healthy, and invulnerable. They avoid thinking about future problems; they are complacent or ignore them based on the phenomenon of optimistic bias. However, the advent of the pandemic also overturned this rule, in light of the fact that the danger from SARS-CoV-2 did not occupy only the press and the media, but took on the dimensions of an individual. The real-life threat was transformed from distant and future into tangible and imminent, giving flesh and blood to the invisible enemy that emerged as a present and immediate danger.

According to the Health Belief Model [49], the degree and likelihood of adopting a health-related behavior (such as the SARS-CoV-2 prevention and control measures) is determined by the perception of the threat, that is, the degree to which the disease is perceived as threatening, and by the perceived susceptibility and severity of the disease. In other words, the children who were taught about the hazard of coronavirus felt and understood that what they learned was not about a theoretical danger or a hypothetical issue, or even an abstract and imaginary danger, but a tangible reality and threat, the consequences of which could affect them and their family as well as the social environment. Even if they did not become sick themselves or members of their immediate family, they experienced significant changes in their daily lives, from the obligation to wear a mask, restrictions on leaving home, to changes in school, and the way they attended classes.

During the pandemic, the students practically recognized their personal involvement with the problem of COVID-19 disease, realized the danger on a personal level, and clarified the meaning of preventive practices, thus mobilizing themselves by willingly applying personal hygiene and protective measures, aiming to protect their own health and defend the safety of their own loved ones, transferring the principles of theory to real life. Opportunities arising from the above observations, which may promote health promotion and disease prevention programs, are supported by the transformation of theoretical knowledge into practice, hypothetical problems into experience, and general instructions into rescue tools; the application of theoretical principles of health education into real-life scenarios, students’ recognition of their personal relationship with the problem of COVID-19 and the development of awareness of the problem on a personal level of risk, which led them to understand the value and meaning of preventive practices; students’ mobilization to willingly implement measures for the protection of their own health and their loved ones; and understanding of preventive interventions in the context of health education, concerning not only the future but also the current risks that are prevented through the adoption of positive health behaviors.

Conclusion

The great historian, Thucydides, characterized the war as a "violent teacher," which creates "stressful" needs and takes away the comfort of life while changing people's behaviors by bringing to the surface features that were left well hidden under the convenience of everyday life during times of peace. This process leads to scraping the glaze of comfort and serenity, setting aside pretexts, and letting the forces of survival emerge. Similarly, we could view the current crisis of the pandemic, which in the field of education put us in front of many obstacles and challenges, but also unfolded hidden possibilities, prospects, and opportunities, acting as a "violent teacher" worth recognizing and exploring. As time passes and the pandemic seems to be fading, we could forget the difficulties we faced, but it is worth keeping and further exploring what they taught us.
